# Supplementary material for: Microglia remodel synapses by presynaptic trogocytosis and spine head filopodia induction
Source: Nat Commun. 2018 Mar 26;9:1228. doi: 10.1038/s41467-018-03566-5 (PMC5964317; doi:10.1038/s41467-018-03566-5)
Supplement: Supplementary file 1 — Supplementary Information(PDF 4849 kb) [file 41467_2018_3566_MOESM1_ESM.pdf]

Microglia remodel synapses by presynaptic trogocytosis and spine head filopodia induction.

Weinhard et al.

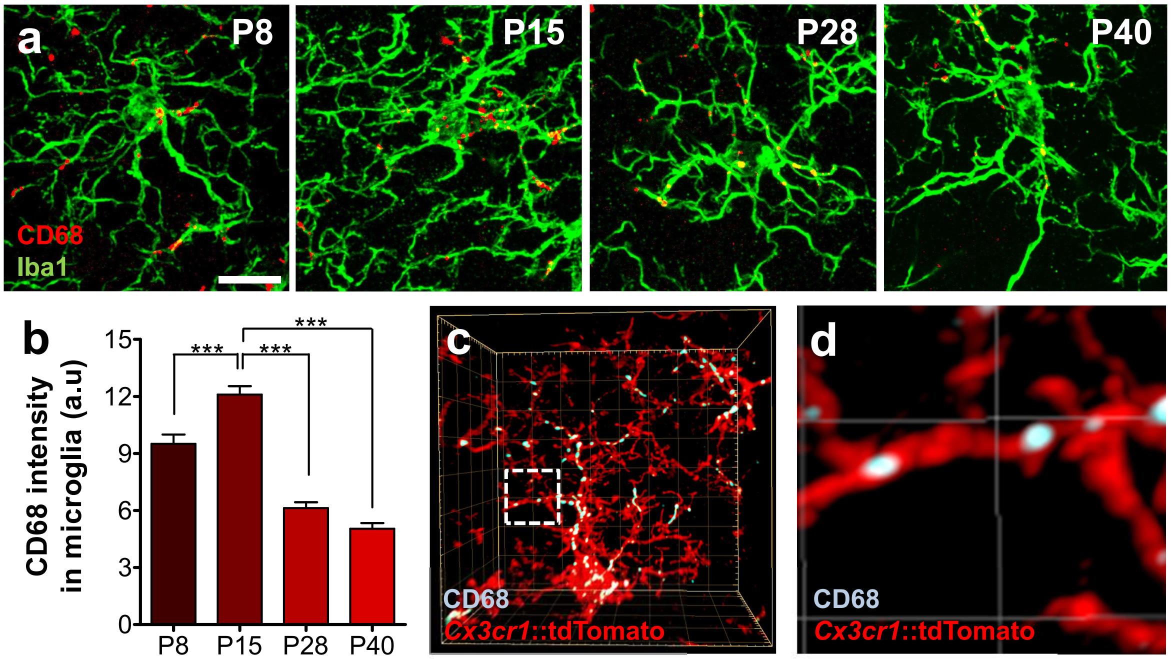

Supplementary Figure 1. Microglia phagocytosis is developmentally regulated. (**a**) Representative images of Iba1-labelled microglia (green) containing phagocytic compartments immunostained for CD68 (red) at P8, P15, P28 and P40 in the *stratum radiatum* of CA1 (scale bar = 10 µm). (**b**) Quantification of CD68 intensity in microglia revealed a peak at P15 in CD68 intensity (*** $P < 0.001$, two-way ANOVA with Bonferroni *post hoc* test, $n = 120$ cells from 6 animals per age group error, error bars are mean + s.e.m). (**c**) Genetically-labelled microglia (red, *Cx3cr1*::CreER; *RC*::LSL-tdTomato) showed similar CD68 expression pattern as Iba1-stained microglia at P15 (enlarged process in [d], grid line increment = 5 µm).

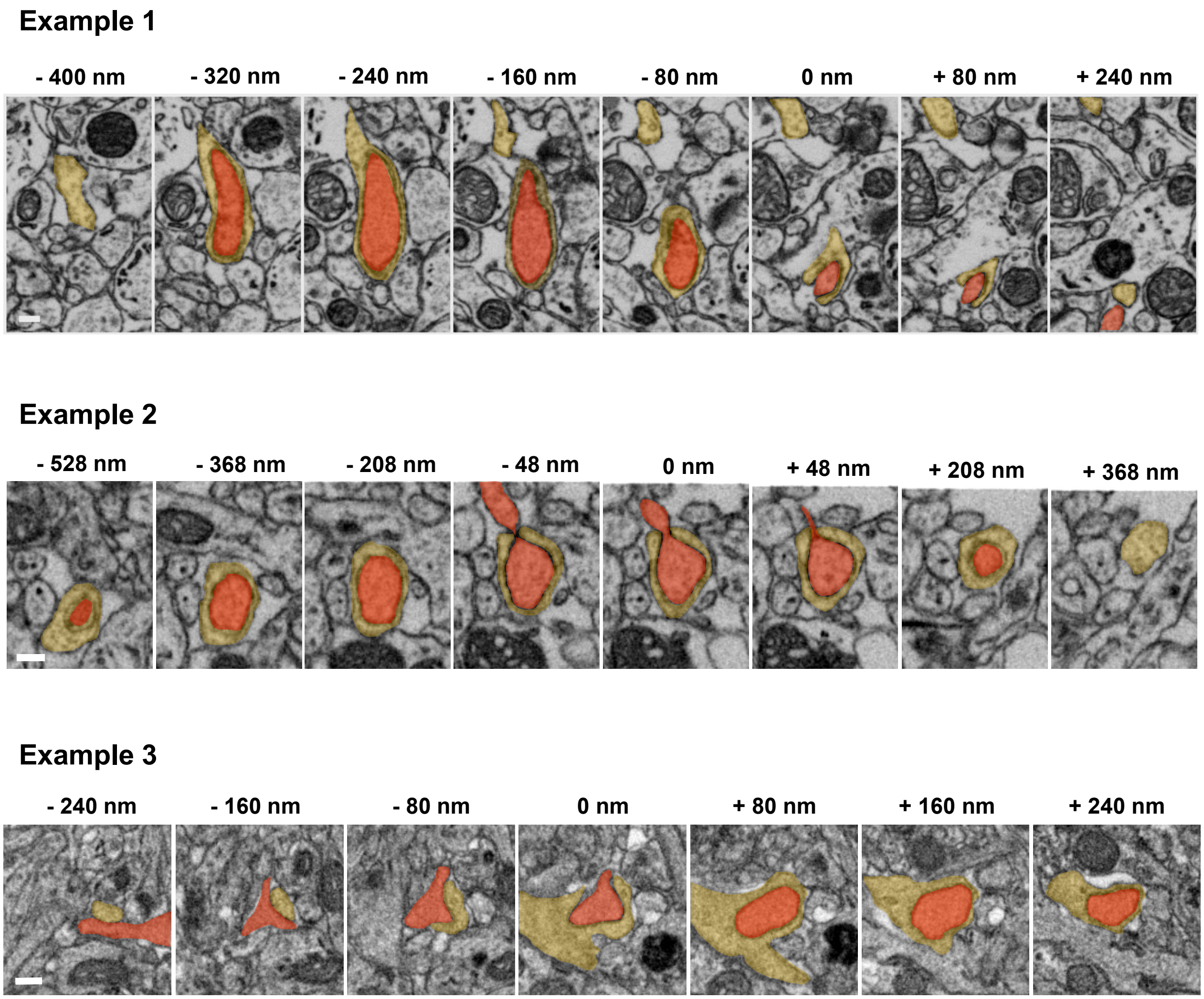

Supplementary Figure 2. Microglia self-engulfment. Representative examples of microglia (yellow) engulfing other microglial processes (orange). In each example, the point of maximum constriction is centred at 0 nm (scale bar = 200 nm).

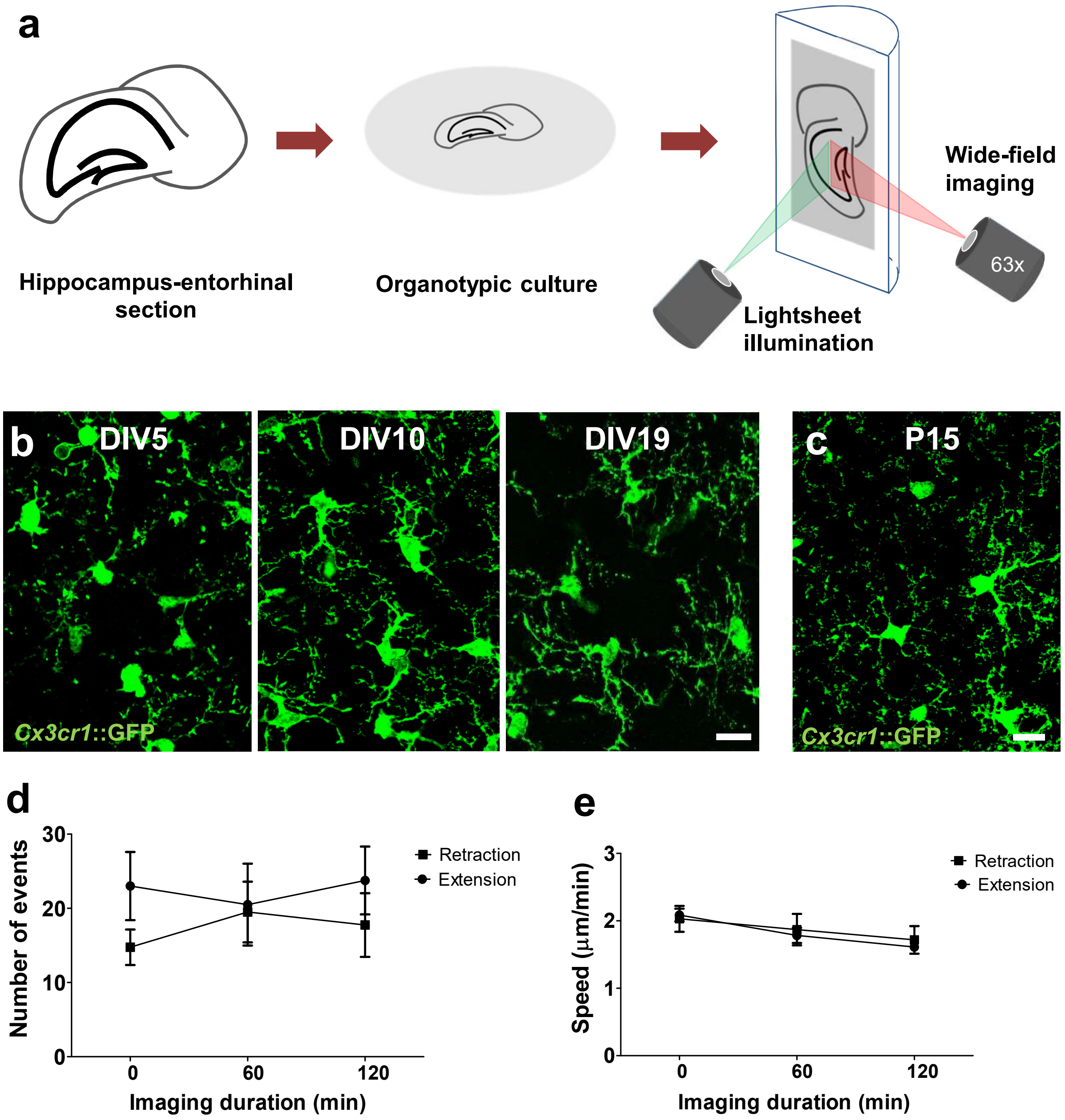

Supplementary Figure 3. Microglia show normal morphology and motility in organotypic cultures. (**a**) Schematic showing the preparation of organotypic hippocampal slice cultures for time-lapse imaging with light sheet microscopy. (**b**) Representative confocal images of microglia (green, *Cx3cr1*::GFP) in fixed slice cultures at 5, 10 and 17 days *in vitro* (DIV). Note the progressive ramification of microglia over time. (**c**) Representative confocal image of microglia in fixed tissue at 15 postnatal days *in vivo* (P15). Note that the morphology is similar to 10-19 DIV slice cultures. (**d**,**e**) Characterization of microglia motility in slice cultures from *Cx3cr1*::CreER; *RC*::LSL-tdTomato mice using time-lapse light sheet imaging (see ***supplementary movie 4***) by quantification of (**d**) number (two-way ANOVA, main effect of time: $F_{2, 12} = 0.22$, $p = 0.81$; main effect of direction: $F_{1, 12} = 0.96$, $p = 0.37$) and (**e**) speed of process extension and retraction events (two-way ANOVA, main effect of time: $F_{2, 12} = 3.48$, $p = 0.06$; main effect of direction: $F_{1, 12} = 0.10$, $p = 0.77$, n = 4 cells from 3 organotypic slice cultures, error bars are mean ± s.e.m, scale bar = 10 µm).

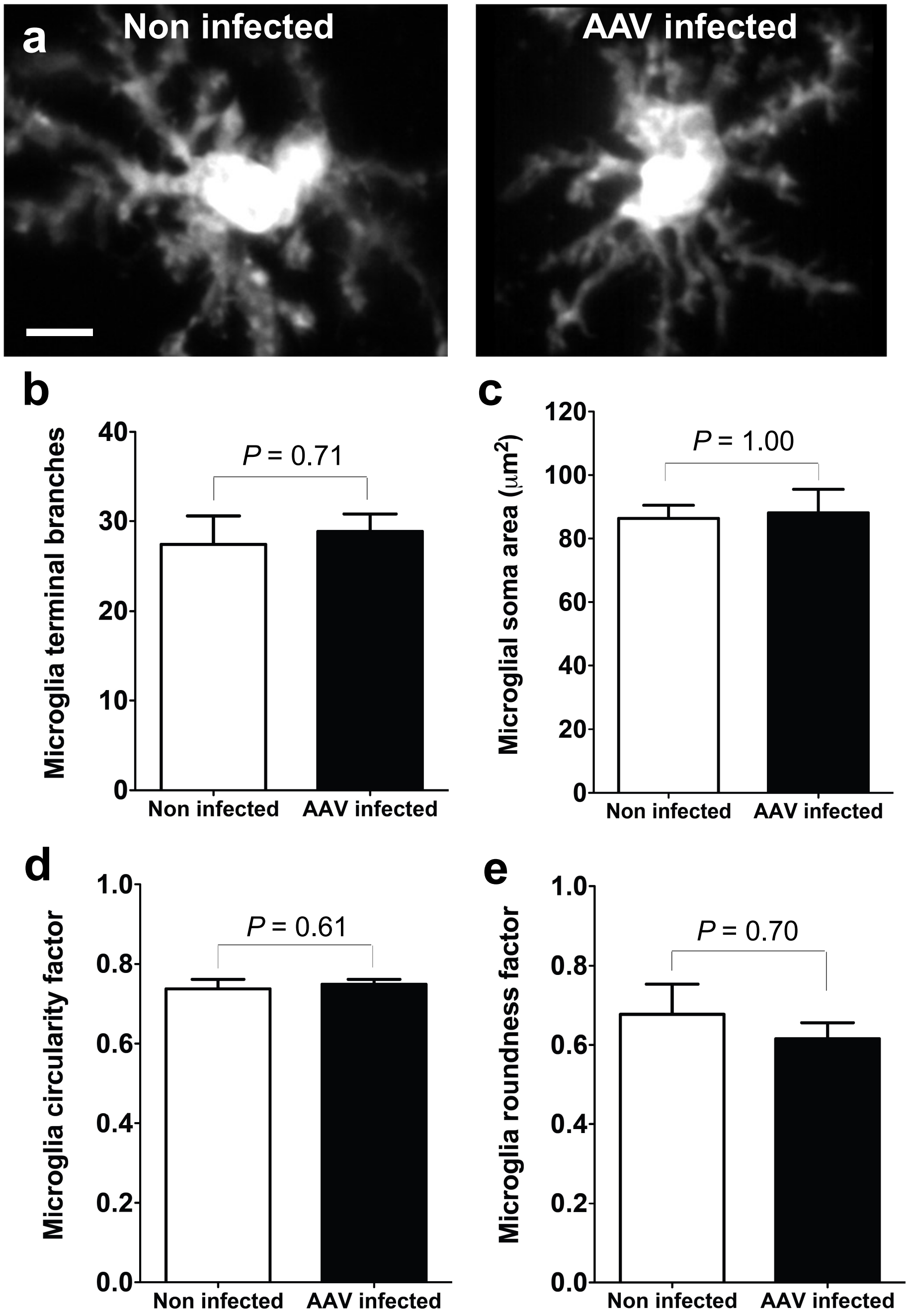

Supplementary Figure 4. No difference in CA1 microglia morphology upon AAV infection of CA3. (**a**) Representative images of microglia in the CA1 region of non-infected versus AAV-infected hippocampal slices two weeks after CA3 infection (stack projection of 40 consecutive optical sections, Δ z = 0.48 µm). No differences were found in microglia (**b**) terminal branches, (**c**) soma area, (**d**) circularity, and (**e**) roundness ([b] two-sided unpaired t-test, [c,d] Two-sided Mann-Whitney test, n = 7 cells from 3 organotypic slice cultures per group, error bars are mean + s.e.m, scale bar = 5 µm).

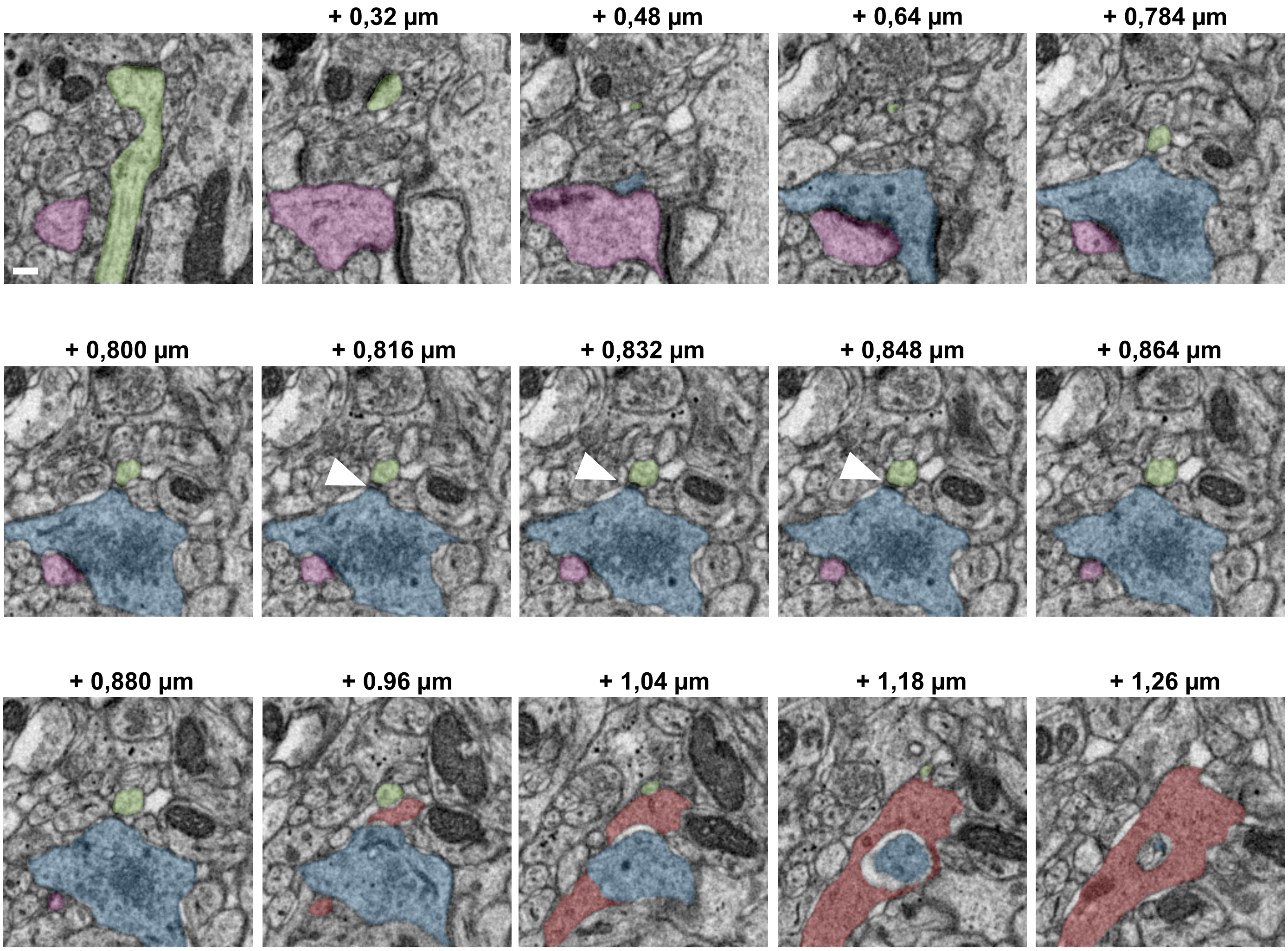

Supplementary Figure 5. FIB-SEM image sequence showing formation an MSB. A spine and its spine head filopodia (green) converging toward a microglial process (red) forms a nascent synapse (white arrows) identified by the presence of a post-synaptic density (PSD) and no presynaptic vesicles at the site of bouton (blue) contact. Note that the contacted bouton already makes a mature synapse with another dendritic spine (magenta), resulting in the formation of a multiple-synapse bouton (MSB, scale bar = 200 nm).

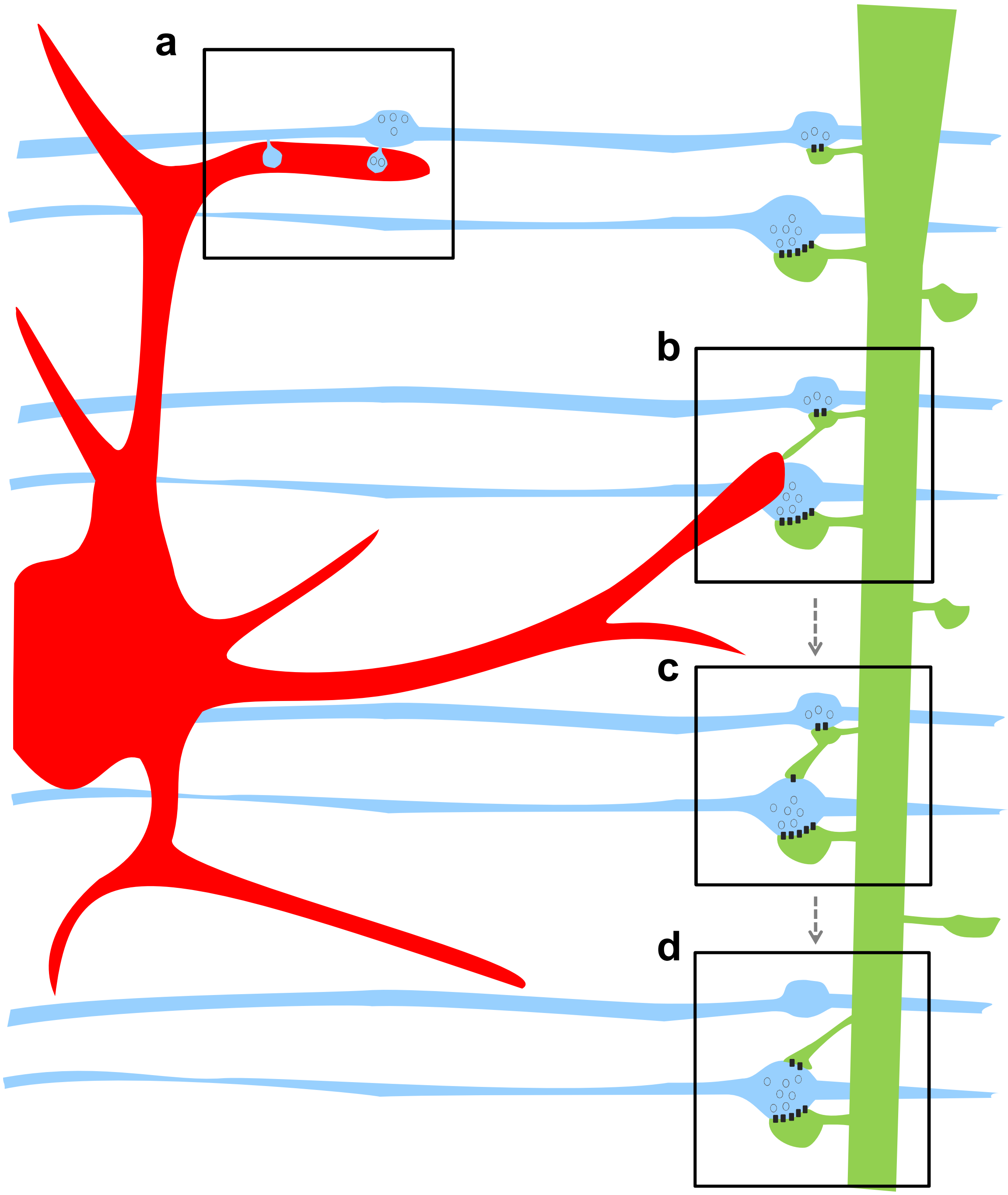

Supplementary Figure 6. Proposed role for microglia in synaptic remodeling. (**a**) Microglia (red) eliminate synaptic material by trogocytosis of presynaptic structures (blue), including both synaptic boutons and axonal shafts. (**b**) Microglia do not eliminate postsynaptic material (green), but frequently induce filopodia formation from mature, synapse-bearing spines. (**c**) The induction of spine head filopodia is occasionally followed by spine switching, a process in which the filopodia facilitates the relocation of the spine head to a new bouton. (**d**) In most cases, this is likely to result in the formation of a multiple synaptic bouton (MSB).
