# Supplementary material for: Microglia remodel synapses by presynaptic trogocytosis and spine head filopodia induction
Source: Nat Commun. 2018 Mar 26;9:1228. doi: 10.1038/s41467-018-03566-5 (PMC5964317; doi:10.1038/s41467-018-03566-5)
Supplement: Supplementary file 3 — Description of Additional Supplementary Files(PDF 48 kb) [file 41467_2018_3566_MOESM3_ESM.pdf]

Description of Additional Supplementary Files

File Name: Supplementary Movie 1
Description: Segmentation and 3D reconstruction of FIB-SEM datasets

File Name: Supplementary Movie 2
Description: FIB-SEM z-stack showing a complete inclusion of presynaptic bouton material inside microglia

File Name: Supplementary Movie 3
Description: FIB-SEM z-stack showing a partial inclusion of axonal material inside microglia

File Name: Supplementary Movie 4
Description: Time-lapse light sheet imaging of Cx3cr1::CreER; RC::LSL-tdTomato microglia

File Name: Supplementary Movie 5
Description: Time-lapse light sheet imaging of presynaptic material intake by microglia

File Name: Supplementary Movie 6
Description: Time-lapse light sheet imaging of presynaptic material intake by microglia in CR3 KO

File Name: Supplementary Movie 7
Description: Time-lapse light sheet imaging of filopodia induction by microglia

File Name: Supplementary Movie 8
Description: Time-lapse light sheet imaging of spine head filopodia induction by microglia
